# Evaluation of free-floating tracheal intubation in weightlessness via ice-pick position with a direct laryngoscopy and classic approach with indirect videolaryngoscopy

**DOI:** 10.1038/s41526-023-00314-y

**Published:** 2023-09-08

**Authors:** Séamus Thierry, François Jaulin, Clément Starck, Philippe Ariès, Jan Schmitz, Steffen Kerkhoff, Cécile Isabelle Bernard, Matthieu Komorowski, Tobias Warnecke, Jochen Hinkelbein

**Affiliations:** 1 Anaesthesiology Department, South Brittany General Hospital, 56100 Lorient, France; 2 Space Medicine Group, European Society of Aerospace Medicine (ESAM), Cologne, Germany; 3 Medical Simulation Centre B3S, 56100 Lorient, France; 4 Laboratoire Psychologie, Cognition, Communication, Comportement, Université Bretagne Sud, 56000 Vannes, France; 5 Sorbonne Medical University, Assistance Publique des Hôpitaux de Paris, Paris, France; 6 Human Factor in Healthcare Association, Group FHS, Paris, France; 7 Anaesthesiology and Intensive Care Department, University Hospital of Brest, 29200 Brest, France; 8 Department of Anaesthesiology and Intensive Care Medicine, University Hospital and Medical Faculty, Cologne, Germany; 9 German Society of Aerospace Medicine (DGLRM), Munich, Germany; 10 Department of Surgery and Cancer, Imperial College London, London, UK; 11 Department of Anaesthesiology, Critical Care, Emergency Medicine and Pain Therapy, Hospital of Oldenburg, Medical Campus University of Oldenburg, Oldenburg, Germany

**Keywords:** Translational research, Risk factors

## Abstract

Long duration spaceflights to the Moon or Mars are at risk for emergency medical events. Managing a hypoxemic distress and performing an advanced airway procedure such as oro-tracheal intubation may be complicated under weightlessness due to ergonomic constraints. An emergency free-floating intubation would be dangerous because of high failure rates due to stabilization issues that prohibits its implementation in a space environment. Nevertheless, we hypothesized that two configurations could lead to a high first-pass success score for intubation performed by a free-floating operator. In a non-randomized, controlled, cross-over simulation study during a parabolic flight campaign, we evaluated and compared the intubation performance of free-floating trained operators, using either a conventional direct laryngoscope in an ice-pick position or an indirect laryngoscopy with a video-laryngoscope in a classic position at the head of a high-fidelity simulation manikin, in weightlessness and in normogravity. Neither of the two tested conditions reached the minimal terrestrial ILCOR recommendations (95% first-pass success) and therefore could not be recommended for general implementation under weightlessness conditions. Free-floating video laryngoscopy at the head of the manikin had a significant better success score than conventional direct laryngoscopy in an ice-pick position. Our results, combined with the preexisting literature, emphasis the difficulties of performing oro-tracheal intubation, even for experts using modern airway devices, under postural instability in weightlessness. ClinicalTrials registration number NCT05303948.

## Introduction

Tracheal intubation is a specialized and invasive airway procedure, used for respiratory support in critically ill patients. This technique allows to secure oxygen supply to the lungs and to protect the airway from gastric aspiration. In terrestrial settings, it is traditionally performed with an operator at the head of the patient, utilizing a direct laryngoscope in one hand to expose the vocal cords, allowing tracheal tube insertion with the other hand. Despite being a life-saving procedure and considered as the gold standard in advanced airway management, performing intubation in conditionals that are less than optimal is dangerous, such as in pre-hospital settings^1,2^ or with operators experiencing a lack of training and practice^3,4^. Intubation is a time-pressured emergency procedure for which failure and side-effects can be life-threatening.

Severe hypoxemia can result from tube insertion delay or failure. Other complications include unrecognized misplacement, secondary dislodgement, laryngeal trauma and hemodynamic response such as bradycardia for examples^5,6^. Defining the scope of this medical procedure in spaceflight conditions, especially under the constraint of weightlessness, has been tested in many medical simulations (underwater and parabolic flight studies)^7–12^.

Recent results confirm that weightlessness is a constraint that impairs the safety of intubation by lowering the probability of first-pass success and delaying the time to provide the first ventilation^10–12^. When performed with a classic direct laryngoscope by novices, oro-tracheal intubation attempts lead to unacceptable high failure rates in both tethered and free-floating positions^8^. In this context, maintaining intubation capabilities in such a difficult environment raised technical and ethical issues^13,14^. But difficulties related to weightlessness could benefit from terrestrial technical, material and conceptual updates.

For example, we can cite the emergence of video-assisted laryngoscopy. These devices allow indirect visualization of the larynx through a camera image from the tip of the laryngoscope to an eyepiece or monitor, on which it is viewed by the operator^15^. This technology outperforms direct laryngoscopy for difficult intubation in numerous hospitals^15–17^ or out-of-hospital studies^18,19^, in novices and experts. These devices also have appealing properties such as a steeper learning curve^15,20^. The benefits of videolaryngoscopy were confirmed in weightlessness in a recent parabolic flight research^21^: in this simulation study where operators and the mannikin were both tethered to the floor, experts and novices experienced high success rates compared to direct laryngoscopy, rising hope for better intubation results in weightlessness. However, many experimental combinations (regarding intubation device, position and operator’s expertise) remained untested in weightlessness and needed to be explored.

In this study, we tested three hypotheses during a parabolic flight campaign in order to refine advanced airway management strategies for spaceflights.

First, we hypothesized that two intubation configurations with a free-floating operator could offer safe outcomes regarding first-pass success. The first experimental condition tested the performance of a conventional direct laryngoscopy in an ice-pick position, the second tested an indirect laryngoscopy with a videolaryngoscope in a classic position at the head of a manikin.

To be considered as a safe approach, each condition had to reach a 95% first-pass success rate. This high percentage is recommended by the ILCOR for tracheal intubation in order to avoid complication and side-effects. Below this threshold, our experimental condition would be considered as unsafe.

Our secondary aim was to confirm the superiority of the first approach using the videolaryngoscope at the head of the manikin over the conventional laryngoscopy in an ice-pick position in microgravity.

Finally, we hypothesized that the ice-pick position success rates would be higher in microgravity than in normogravity, as this environmental condition could offer more degrees of movement for this approach. This may seem counter-intuitive as microgravity is often depicted as a disabling environment regarding the execution of emergency procedures, but curiously microgravity has already proven to ease an intubation approach in a previous study^21^. We wanted to analyzed this effect in our study, as it could potentially open a new conception in ergonomic positioning for weightlessness intubation.

## Results

### Comparison of the two techniques in weightlessness

The success rate for intubation with video laryngoscopy in a free-floating position was higher than the ice-pick approach with a direct laryngoscopy (generalized linear mixed-effects model (GLMM), OR = 1,27; 95%CI = [1,07, 1,51]; *p* = 0,005). No statistical difference was highlighted regarding time and confidence score (Table 1).

**Table 1:** comparison of the two intubation approaches in microgravity.

	Ice-pick direct laryngoscopy	Video laryngoscopy	Statistical test: GLMM model
Number of attempts	45	45	
Success, *n* (%)	15 (33.3%)	26 (57.8)	*p* = 0,005
Time (median, IQR)	22.0 (18.0, 24.0)	20.0 (13.0, 24.0)	*p* > 0,05
Confidence score (median, IQR)	−5.0 (−8.0, 5.0)	10 (−10, 10)	*p* > 0,05

### Comparison of ice-pick intubation in normogravity and in weightlessness

Results are shown in Table 2. Ice-pick intubation had in both conditions low success scores for intubation (microgravity 15/45 (33.33%) versus normogravity 21/40 (52.5%). With the GLMM model, performing ice-pick intubation is significantly more successful in normogravity than in microgravity (OR = 1.3; 95%CI = [1.11, 1.52]; *p* = 0.001). There was no statistical difference in intubation duration and confidence score between the two gravity conditions.

**Table 2:** Comparison of the ice-pick approach in normogravity and microgravity.

	Ice-pick microgravity	Ice-pick normogravity	Statistical test: GLMM model
*n*	45	40	
Success, *n* (%)	15/45 (33.3)	21/40 (52.5)	*P* = 0,001
Time, mean (SD)	20.5 (4.3)	18.1 (5.4)	*p* > 0,05
Confidence, mean (SD)	−2.0 (7.0)	0.5 (8.3)	*P* > 0,05

### Was there a fatigue or learning effect during the experiment?

The logistic regression models failed to identify learning effects in both intubation conditions in microgravity: with video laryngoscopy (OR = 1.14; 95%CI = [−0.284, 0.559]; *p*-value = 0.5215) and using the ice-pick technique (OR = 1.29; 95%CI = [0.82, 2.02]; *p*-value = 0.2613). However, the number of parabolas might have been too low to develop a learning effect.

### Did the confidence score predict the success of an intubation?

Confidence reliably predicted the success of intubation, for both techniques and under both gravity conditions (OR = 1.46; 95%CI = [1.27, 1.68], *p*-value < 0.001).

## Discussion

The main finding in our study is that neither of the two free-floating configurations tested were compatible with intubation safety standards. As a reminder, for airway management of terrestrial cardiac arrest in pre-hospital settings, the International Liaison Committee on Resuscitation (ILCOR) recommends that “*only systems that achieve high tracheal intubation success should be used”*. The expert consensus defined a high success score as greater than 95% with up to 2 intubations attempts^22^.

Regarding the ice-pick intubation with a direct laryngoscope, this approach remained difficult even for trained operators, as they had a poor and unacceptable first pass success rate (33%) and a low confidence score with this technique. This success rate among experts is similar to those obtained under other free-floating configurations^8,11,12^.

In the second experimental condition, experts used videolaryngoscopy in the free-floating condition and achieved better success scores than under the icepick strategy. But with a first pass success rate of only 57%, mostly due to stabilization issues, this free-float approach doesn’t meet the required standards for a safe utilization. Our results confirm that free-floating intubation is dangerous for the patient, even when experts equipped with modern intubation devices perform the procedure. In a previous study^21^, we suggested that video laryngoscopy may be more suitable than direct laryngoscopy when operator and manikin were tethered to the floor, which could be similar to a planned intubation configuration (for a semi-urgent surgical procedure, i.e.). Video laryngoscopy tackled to some extent the expertise issue, as untrained operators reached high success scores^21^, but this benefit seems to vanish in free-floating conditions.

To put these results into perspective, it is important to understand the issues related to maintaining airway management capabilities during space missions. From a theoretical point of view, it seems logical to propose advance life support protocols during space mission, as astronauts live and work in a dangerous environment, far from any definitive medical facility^23^. They are exposed to several health hazards, including potential hypoxemic emergencies that would require respiratory support. Many conditions could lead to an abolished respiratory drive (toxic coma, cardiac arrest, general anesthesia) or severe pulmonary alveolocapillary membrane dysfunctions (smoke inhalation, foreign body inhalation, burns). As a reminder, The *International Space Station* is equipped with airway management capabilities, including a direct laryngoscope to perform tracheal intubation, available in a dedicated respiratory kit^24^. Interestingly, to this day, no advanced airway procedure has been necessary during Low Earth Orbit missions. This data reflects the success of safety policies and space medicine, implementing drastic preventive actions and countermeasures to control medical risks in orbital stations. In addition, astronauts have therapeutics options to stabilize early-stage lung conditions (i.e., antibiotics for pulmonary infection) and to rapidly return to Earth within a few hours in case of a looming respiratory threat, leaving an on-board intubation to a worst-case scenario with a very low probability event^25,26^.

The other important point is that the future of crewed spaceflight is shifting to new medical risk configurations. Firstly, planification of long duration exploratory missions to the Moon or Mars will set a group of four to six professional astronauts in a dangerous and remote environment, far from any definitive medical care facility and no immediate evacuation option back to Earth. Potential medical contingencies are numerous, as highlighted in prediction models provided by space agencies^27–30^, with some scenarios requiring the need for oxygen support, artificial ventilation and advanced airway management skills^31,32^. We can cite examples of cardiac arrest, severe decompression sickness, pneumoniae related to exposition to irritative planetary dust^33,34^ or abdominal surgery occurring during the mission, either in weightlessness (during interplanetary travel) or partial gravity (at the Moon or Mars surface)^35–37^.

Secondly, the development of commercial sub-orbital and orbital spaceflights may open this environment to individuals traditionally ruled out by professional astronaut medical selection^38^ in this configuration, individuals with potential pre-existing chronic and stabilized conditions may experience exposure to the space environment: “patients” would become “astronauts” with an increased background risk for the need of airway support during the flight^39^.

Unfortunately, human spaceflight combines most (if not all) of the risk factors for intubation’s failure and complications, generating legitimate questions about its availability in such an austere environment. These factors emerge from three sources: the astronaut-patient, the astronaut-caregiver and the space environment itself, especially the constraint of weightlessness. Regarding the astronaut-caregiver for example, long term exposure to spaceflight and microgravity can affect the safety of intubation. As examples, we can cite decline in sensorimotor, orientation and cognitive performance^40–44^, and loss of vision^45^.

Finally, in most configurations, tracheal intubation would likely be performed by a non-expert astronaut, with minimal or no pre-existing training facing a risk of competency fading. Skill maintenance is problematic, with the risk of fading dexterity after an extended absence of practice, even after an adequate initial training^46^.

These findings regarding intubation’s safety under weightlessness refine and reinforce the validity of recent cardiopulmonary resuscitation for spaceflight guidelines^14^. In this work, a group of experts proposed a data-driven and stepwise approach of advanced airway management. These recommendations state that, if intubation high success rates can’t be achieved due to the environmental hazards of spaceflight, it is recommended to prefer alternative devices such as a supraglottic airway (SGA) in first intention under weightlessness especially if operators are free-floating or novice to airway management.

These recommendations fit with the topic of pre-hospital airway management, which has been the source of many high-quality papers^22^,^47–49^. As with difficult airway access scenarios on Earth^50^, in a weightlessness environment (in parabolic flights or underwater studies), insertion of supraglottic airway is easier than tracheal intubation, even under free floating position and with minimal pre-existing expertise^9–12^. Therefore, they could be advantageous as they allow a quicker time to ventilation compared to tracheal intubation, generating equivalent outcomes regarding airway management during out-of-hospital cardiac arrest^51^.

But supraglottic airway could have disadvantages compared to tracheal intubation. Astronauts may be considered having a full stomach due to reduced gastro-intestinal motility in space^52^, theoretically exposing them to silent aspiration. Further studies are warranted to clarify the risk of regurgitation and aspiration in space. Thus, SGA may be suitable as an initial airway management device^53^, but they do not represent a definitive airway device. Moreover, secondary dislodgments are more frequent with SGA than tracheal tubes, especially during transport phases^51^, although, at this day, aeromedical evacuation of an intubated astronaut is not possible due to many constraints.

Some limitations can be highlighted in this study. First, all participants had a previous experience of real or simulation intubation and were considered as experts. This high-level of intubation expertise was shown to be associated with high success in prehospital intubation^54^ but may not reflect the real profile of the on-board caregiver in future space missions^55^. On the contrary, the relatively short weightlessness period (22 s) allowed for each attempt, combined with the presence of very difficult intubation settings (cervical collar^56^) may be a possible confounding factor regarding high failure rates.

Another limitation is that access to microgravity platforms such as parabolic flights is rare, inherently limiting the number of experimental conditions available for such simulation studies^57^.

Regarding the tested videolaryngoscope, it is important to remember that videolaryngoscopy regroups a heterogenous class of different devices^58^, each of them proposing specific technological approaches to visualize and expose the airway through design and blade structure (hyper-angulated or not, channeled or not). Each setting could therefore influence the final success rate. We did not conduct a videolaryngoscopy comparison study due to experimental time limitations. We used the McGrath^®^ videolaryngoscope with a non-angulated blade that is very similar to the MacIntosh conventional laryngoscope blade, but the use of a videolaryngoscope with a hyper-angulated blade may have offer higher success rates in microgravity.

The McGrath^®^ videolaryngoscope can theoretically be used either as an indirect, video-assisted device or as a direct laryngoscope, which could be valuable in a worst-case scenario in which the screen became non-functional. But this direct laryngoscopy functionality was not tested in our study. As a reminder, the latter is associated with more difficult intubation and worse glottic view than a conventional laryngoscope in the literature^59^.

Finally, we did not test video-laryngoscopy in the ice-pick position, which deserves to be tested in a further study and could bring interesting data about indirect laryngoscopy’s range of validity in weightlessness. Previous studies in normogravity regarding videolaryngoscopy in ice-pick position highlighted inconsistent results regarding its safety^18,60^. Nevertheless we believe its exploration under weightlessness could be valuable as some studies were positive with a variety of devices^61–63^.

Regarding the outcome, intubation attempts were considered as failed in case of unipulmonary ventilation. This outcome may be considered as restrictive, since unipulmonary ventilation may be sufficient for providing a basic level of oxygenation while also protecting the lungs against aspiration once the balloon is inflated. As post-intubation auscultation has to occur anyway, correction of the tube position could be achieved in a reasonable amount of time after intubation. But this correction could become dangerous in a space environment: the noisy environment could disturb lung auscultation, and could lead to accidental extubation during tube position correction. We therefore excluded selective intubation in order to avoid overestimating the reliability of the tested condition.

Finally, this study only focuses on a limited segment of a complete intubation procedure. The success of intubation relies on a delicate interplay of expertise, reliable device, associated tasks management (sedation, drug preparation) and non-technical skills. The management of a critically ill patient requiring intubation is not limited to the laryngoscopy technique. The context of any pre-hospital intubation scenario includes advanced technical and non-technical skills. Mastering alternative oxygenation strategies^64^, correct drug storage and preparation, management of early and late intubation side effects, sustained oxygen delivery and optionally aeromedical evacuation with continuous care^65^ and extubation readiness criteria are some of the unresolved questions raised by an airway emergency in space. To date, no evacuation of a critically ill or intubated patient is possible onboard the current space vehicles^26^ and “space ambulances”, allowing evacuation of a critical ill from Low Earth Orbit to a Definitive Medical Care Facility once evoked, were abandoned^66^. Many ethical issues related to anesthesia and critical care provisions in austere environments are still open, far beyond technical consideration related to the sole criteria of laryngoscopy^23,32,67–70^.

In conclusion, under free floating conditions, we found that using direct laryngoscopy with the ice-pick position for intubation is not a safe approach. Free-floating videolaryngoscopy in a classic position (at the head of the manikin) shows superiority over the ice-pick position but also fails to reach the minimum standards regarding first pass success among trained physicians. Combined with existing literature, this result suggests that performing endotracheal intubation under postural instability with conventional direct laryngoscopy is very difficult, even for trained experts. We suggest equipping current and further space missions with a video-laryngoscope. Intubation should only be performed if the three following conditions are reunited: the procedure has to be performed under restrained conditions (i.e planned intubation), with a videolaryngoscope and has to be led by a trained operator. Any other configuration imposes operators to switch to supraglottic devices for initial advanced airway management. Promising lines of research regarding this topic could focus on devices (robotic intubation^71,72^ or new video-laryngoscope designs^63^) but also on procedures assistances such as artificial intelligence for clinical decision support in austere environment^73,74^.

## Methods

The study took place onboard the Novespace Airbus A310 Zero-G during the 57^th^ French National Space Agency (Centre National d’Etudes Spatiales -CNES) parabolic flight campaign. We conducted a non-randomized, controlled, cross-over study comparing two intubation techniques under two gravity conditions. The ice-pick intubation was tested with a conventional direct laryngoscope in weightlessness and in normogravity. This was compared to a second free-floating position, with the operator using indirect videolaryngoscopy at the head of a manikin. This study was authorized by the CNES – French National Space Agency ethical committee and registered at ClinicalTrials.gov (NCT05303948).

### The ice-pick position with a direct laryngoscope

**Fig. 1:**
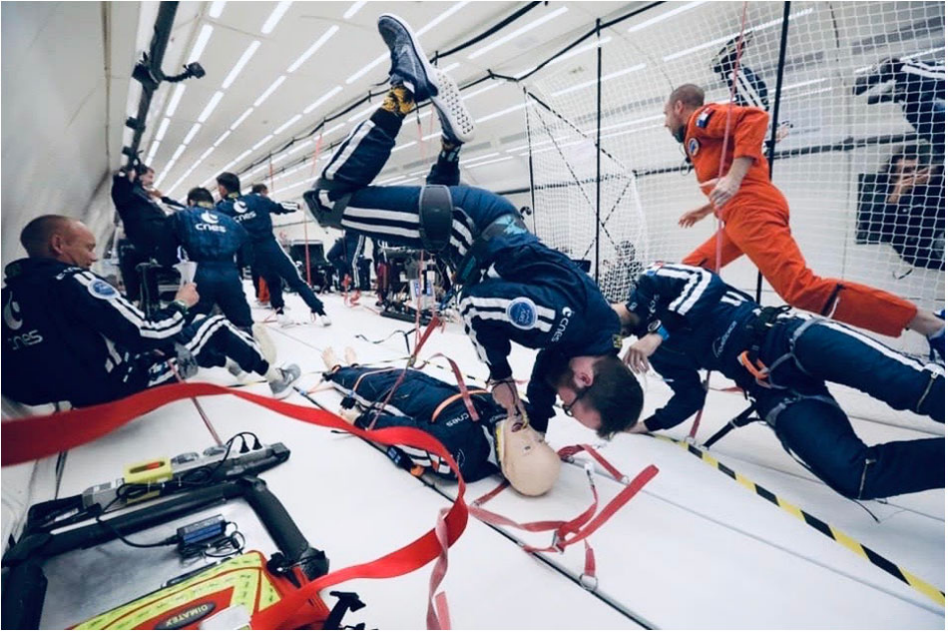
Ice-pick position with a classic laryngoscope in simulated microgravity. (Credit : Alexis Rosenfeld, Novespace© and CNES. All participants consented to the publication of the photographs).

Our first experimental condition was performing intubation with a direct laryngoscope under the “ice-pick” or “face-to-face” position (Fig. 1). This alternative position is the last ergonomic configuration in which direct laryngoscopy has not yet been evaluated in weightlessness. In the literature, direct laryngoscopy was always tested with the subject at the head of the manikin, restrained of free-floating. This unconventional approach can become useful in pre-hospital settings^75,76^, in case of restricted access to the patient^77^, for example in a confined or narrow recess such as in a car wreck.

This technique could present some ergonomic advantages in a weightlessness environment. It could allow the caregiver to have a counter surface (the patient’s chest) to stabilize himself with his knees while performing the procedure, and potentially offering more biomechanical strengths to expose the glottic plan by using glenohumeral retraction forces rather than protraction^78,79^. Additionally, preparation and setup for airway management could also compete with some specific chest compression positioning in weightlessness, such as the “reverse bear hug” technique, performed by operator behind the patient^26,80^.

### Free-floating videolaryngoscopy

**Fig. 2:**
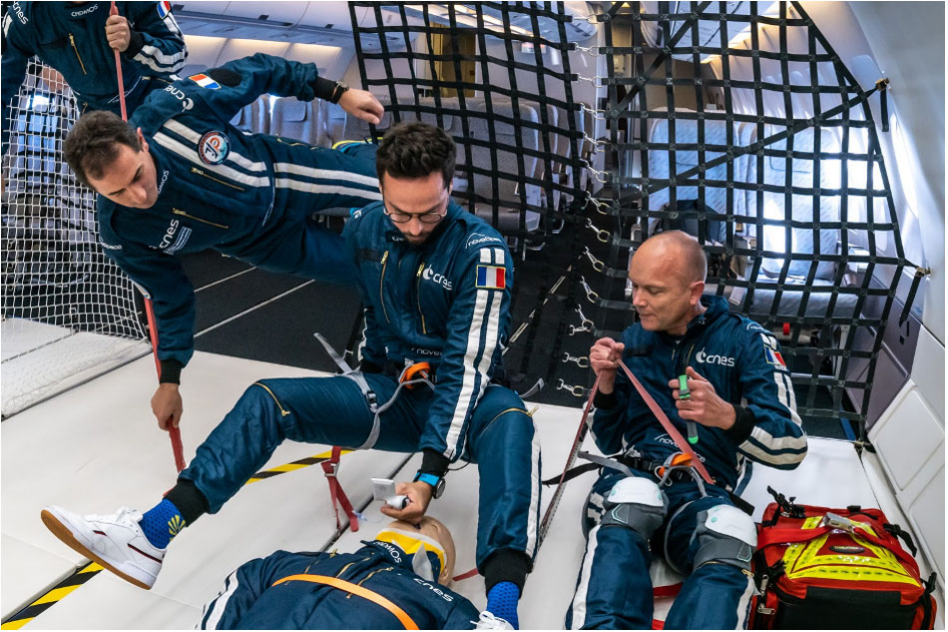
Free-floating position at the head of the manikin with a video laryngoscope. On the right, an assistant operator measuring time, success and recording the operator confidence score. (Credit : Alexis Rosenfeld, Novespace© and CNES. All participants consented to the publication of the photographs).

The second uncertainty concerned the performance of indirect videolaryngoscopy with an operator subject to postural instability (Fig. 2). The positive results obtained by videolaryngoscopy described previously were obtained under restrained positions^21^ and needed to be challenged with additional degrees of movement. In the case of a sudden on-board emergency requiring advanced airway management, intubation could be performed by a free-floating operator, without having time to restrain themselves to a dedicated stretcher.

### Participants

Nine trained subjects, eight male and one female participated in the experiment. Seven subjects had a daily practice of intubation, two subjects had an intubation experience of over 20 procedures in a simulation center. Participants performed laryngoscopy in ice-pick position 85 times (45 in weightlessness, 40 in normogravity) and video laryngoscopy in a free-floating position 45 times (all in weightlessness), resulting in a total of 130 intubation attempts. All participants were volunteers and provided written informed consent for the research. The subjects were medically certified fit to fly and given subcutaneous scopolamine around 2 h before each flight to prevent motion sickness. Of nine subjects, four experienced zero gravity for the first time.

### Simulating microgravity

Each parabola onboard the Airbus A310 Zero-G consisted of about 22 s of hypergravity at 1.8 g, during which the plane accelerated upward at a 45-degree angle, then about 22 s of free falling in the parabola (simulated microgravity), followed by another period of hypergravity corresponding to the recovery of the plane to the starting altitude.

### Mannequin and equipment

Tracheal intubation was performed using a high-fidelity full body difficult airway training mannequin (SimMan ALS; Laerdal International, Stavanger, Norway) configured for difficult intubation. In space, astronauts experience cephalic congestion (the “*puffy-face syndrome*”) because of fluid redistribution from the lower half of the body^44,81^. To reflect this, the tongue of the mannequin was inflated. We also restricted further cervical motion with a rigid collar (Stifneck Select, Laerdal International). Insertion of a 7.0 mm cuffed oro-tracheal tube was attempted using either conventional direct laryngoscopy in an ice-pick position or indirect laryngoscopy with a McGrath^®^ video laryngoscope (McGrath^®^ model, Covidien™, Medtronic™), both fitted with a Macintosh non-angulated size 3 blade. The operator was loosely strapped, allowing postural instability. Complete free-floating during the experiment was not permitted by flight engineers due to security reasons.

### In-flight experimental set up

Three flights were conducted, each offering 30 parabolas during which 30 weightlessness tracheal intubations were attempted (15 in ice-pick position, 15 in classic position with video laryngoscopy). After each flight, 30 normogravity attempts were performed inside the plane stationary on the ground, following the same experimental setup and sequence, for a total of 130 intubation attempts. Each flight boarded three operators that sequentially performed five consecutive intubation attempts using either direct laryngoscopy in ice-pick position or video laryngoscopy before switching roles and devices. The manikin was tethered to the cabin floor. In ice-pick position, the operator started at the feet of the mannequin, whereas he/she was sitting at the at the head of the mannequin in the classic configuration. A third assistant timed the attempts and recorded all data on a paper chart.

### Outcomes

Bi-pulmonary ventilation success was assessed by the intubating operator performing chest auscultation and by measurement of a tidal volume on the mannequin’s electronic sensors. Selective side intubation was considered as failure. The duration of each attempt was recorded and ended with vocal confirmation of tube placement from the intubating operator or at the end of the parabola. Finally, a subjective score of confidence in the correct tube placement was recorded after each attempt and ranked from minus ten (complete certainty about failure) to plus ten (complete certainty about success). A score of zero was pronounced by the operator when he/she had maximum uncertainty about success or failure.

### Intubation sequence in weightlessness

Each subject alternatively performed five consecutive, non-randomized intubation attempts by using ice-pick conventional direct laryngoscopy or video laryngoscopy before switching position and device. One intubation attempt was performed per parabola. Each parabola started with the intubating operator holding the device in his/her right hand. After entering weightlessness, the intubating operator inserted the device in the manikin’s mouth and attempted to expose the glottis. The assistant operator handed over the endotracheal tube to the intubating operator, who then tried to insert it in the trachea. Each attempt ended either after tube insertion or at the end of the free-floating period. The time between parabolas (around 90 s) was used to check the position of the tube and to reset the experimental setup by the assistant operator. The intubation operator verbalized his confidence score, then inflated the cuff and auscultated the chest during manual bag ventilation.

### Intubation sequence in normogravity

For ice-pick intubation, a twin study was performed on the same day by the same crew after the flight and took place on board the plane stationary on the ground. Normogravity records occurred after the weightlessness records in order to avoid a training effect. Experimental settings, time for intubation and sequences were identical to the in-flight study in order for gravity to be the only substituting variable. Intubation with video laryngoscopy was not tested, as it was already tested in a previous study^21^.

### Statistical analysis

No statistical power calculation was conducted prior to the study. The sample size was dictated by the number of parabolas available, and the number of conditions to be tested. 95% confidence intervals (CI) were provided on percentages and means with *p* < 0.05 as criterion of statistical significance.

### Logistic regression

For each intubation technique, a logistic regression was performed to highlight a potential fatigue or learning effect (as operators performed attempts in a row) and to test whether the confidence score could predict the success of the intubation.

### Generalized linear mixed-effects models

Regarding success scores, relations between different outcomes and variables were analyzed with generalized linear mixed-effects models (GLMM), an extension to the generalized linear model (GLM) which takes into account random effects. These models are traditionally used for longitudinal data, such as measurements within successive parabolas. The choice of this model is based on the possible learning effect that applied to each subject during the in-flight intubation sequences. Repeating procedures exposes subjects to learning effects which could possibly exclude any hypothesis of independence between successive measurements, regardless of the intubation technique used. Intubation times were specifically measured based on the exclusion of trials that lasted more than the 22 s parabola period. Data was processed with Python (library *Statsmodels*).

### Reporting summary

Further information on research design is available in the Nature Research Reporting Summary linked to this article.

### Supplementary information

Reporting Summary

## Data Availability

All relevant data are available from S.T. on request.

## References

[1] Sumann G (2020). Multiple trauma management in mountain environments - a scoping review. Scand. J. Trauma. Resusc. Emerg. Med..

[2] Helm M, Hossfeld B, Schafer S, Hoitz J, Lampl L (2006). Factors influencing emergency intubation in the pre‐hospital setting – a multicentre study in the German Helicopter Emergency Medical Service. Br. J. Anaesth..

[3] Bossers SM (2015). Experience in Prehospital Endotracheal Intubation Significantly Influences Mortality of Patients with Severe Traumatic Brain Injury: A Systematic Review and Meta-Analysis. PLoS One.

[4] Bernhard M, Mohr S, Weigand MA, Martin E, Walther A (2012). Developing the skill of endotracheal intubation: implication for emergency medicine. Acta Anaesthesiol. Scand..

[5] Cook T, Macdougall-Davis S (2012). Complications and failure of airway management. Br. J. Anaesth..

[6] Mort TC (2004). Emergency tracheal intubation: complications associated with repeated laryngoscopic attempts. Anesth. Analg..

[7] Keller C (2000). Airway management during spaceflight: a comparison of four airway devices in simulated microgravity. Anesthesiol.

[8] Groemer GE (2005). The Feasibility of Laryngoscope-Guided Tracheal Intubation in Microgravity During Parabolic Flight: A Comparison of Two Techniques. Anesth. Analg..

[9] Rabitsch W (2006). Airway management with endotracheal tube versus Combitube during parabolic flights. Anesthesiology.

[10] Warnecke T (2019). Airway management in microgravity: A systematic review. Acta Anaesthesiol. Scand..

[11] Warnecke T (2021). Time to ventilation and success rate of airway devices in microgravity: A randomized crossover manikin-trial using an underwater setting. Acta Anaesthesiol. Scand..

[12] Hinkelbein J (2021). Using supraglottic airways by paramedics for airway management in analogue microgravity increases speed and success of ventilation. Sci. Rep..

[13] Roan RM, Boyd GL (2007). Prediction of a low success rate of astronauts in space in performing endotracheal intubation. Anesthesiology.

[14] Hinkelbein J (2020). Cardiopulmonary resuscitation (CPR) during spaceflight - a guideline for CPR in microgravity from the German Society of Aerospace Medicine (DGLRM) and the European Society of Aerospace Medicine Space Medicine Group (ESAM-SMG). Scand. J. Trauma. Resusc. Emerg. Med..

[15] Lewis SR (2017). Videolaryngoscopy versus direct laryngoscopy for adult patients requiring tracheal intubation: a Cochrane Systematic Review. Br. J. Anaesth..

[16] Hinkelbein J, Iovino I, De Robertis E, Kranke P (2019). Outcomes in video laryngoscopy studies from 2007 to 2017: systematic review and analysis of primary and secondary endpoints for a core set of outcomes in video laryngoscopy research. BMC Anesthesiol..

[17] Higgs A (2018). Guidelines for the management of tracheal intubation in critically ill adults. Br. J. Anaesth..

[18] Asai T (2009). Tracheal intubation with restricted access: a randomised comparison of the Pentax-Airway Scope and Macintosh laryngoscope in a manikin. Anaesthesia.

[19] Boedeker BH, Berg BW, Bernhagen M, Murray WB (2009). Endotracheal intubation in a medical transport helicopter comparing direct laryngoscopy with the prototype Storz CMAC videolaryngoscope in a simulated difficult intubating position. Stud. Health Technol. Inform..

[20] Konrad C, Schüpfer G, Wietlisbach M, Gerber H (1998). Learning manual skills in anesthesiology: Is there a recommended number of cases for anesthetic procedures?. Anesth. Analg..

[21] Starck C (2020). Tracheal intubation in microgravity: a simulation study comparing direct laryngoscopy and videolaryngoscopy. Br. J. Anaesth..

[22] Soar J (2019). 2019 International Consensus on Cardiopulmonary Resuscitation and Emergency Cardiovascular Care Science with Treatment Recommendation. Circulation.

[23] Komorowski M, Fleming S, Mawkin M, Hinkelbein J (2018). Anaesthesia in austere environments: literature review and considerations for future space exploration missions. npj Microgravity.

[24] Barratt M (1999). Medical support for the international space station. Aviat. Space Environ. Med..

[25] Bacal K, Beck G, McSwain NE (2004). A Concept of Operations for Contingency Medical Care on the International Space Station. Mil. Med..

[26] Nowadly CD, Trapp BD, Robinson SK, Richards JR (2019). Resuscitation and evacuation from low Earth orbit: a systematic review. Prehosp. Disaster Med..

[27] Horneck G (2006). HUMEX, a study on the survivability and adaptation of humans to long-duration exploratory missions, part II: Missions to Mars. Adv. Space Res..

[28] Walton ME, Kerstman EL (2020). Quantification of Medical Risk on the International Space Station Using the Integrated Medical Model. Aerosp. Med. Hum. Perform..

[29] Swaffield TP, Neviaser AS, Lehnhardt K (2018). Fracture risk in spaceflight and potential treatment options. Aerosp. Med Hum. Perform..

[30] Billica RD (1996). Perception of the medical risk of spaceflight. Aviat. Space Environ. Med..

[31] Komorowski M, Neuhaus C, Hinkelbein J (2015). Emergency medicine in space. Notf.+ Rettungsmedizin.

[32] Komorowski M, Thierry S, Stark C, Sykes M, Hinkelbein J (2021). On the challenges of anesthesia and surgery during interplanetary spaceflight. Anesthesiology.

[33] Prisk GK (2019). Pulmonary challenges of prolonged journeys to space: Taking your lungs to the moon. Med J. Aust..

[34] Patel ZS (2020). Red risks for a journey to the red planet: The highest priority human health risks for a mission to Mars. npj Microgravity.

[35] Belobrajdic B, Melone K, Diaz-Artiles A (2021). Planetary extravehicular activity (EVA) risk mitigation strategies for long-duration space missions. npj Microgravity.

[36] Panesar SS, Ashkan K (2018). Surgery in space. Br. J. Surg..

[37] Anderton R, Posselt B, Komorowski M, Hodkinson P (2019). Medical considerations for a return to the Moon. Occup. Med..

[38] Stepanek J, Blue RS, Parazynski S (2019). Space medicine in the era of civilian spaceflight. N. Engl. J. Med..

[39] Smith, T. G. Buckey, J. C. Anaesthetists and aerospace medicine in a new era of human spaceflight. *Anaesthesia*10.1111/anae.15580 (2022). 10.1111/anae.15580 34496029

[40] Young LR, Oman CM, Watt DG, Money KE, Lichtenberg BK (1984). Spatial orientation in weightlessness and readaptation to earth’s gravity. Science.

[41] White O (2016). Towards human exploration of space: the THESEUS review series on neurophysiology research priorities. npj Microgravity.

[42] Harris LR, Jenkin M, Jenkin H, Zacher JE, Dyde RT (2017). The effect of long-term exposure to microgravity on the perception of upright. npj Microgravity.

[43] Fiore, S. Wiltshire, T. Sanz, E. Pajank, M. Critical team cognitive processes for long-duration exploration missions - final report 2015. NASA/TM-2015-218583.

[44] Tanaka K, Nishimura N, Kawai Y (2017). Adaptation to microgravity, deconditioning, and countermeasures. J. Physiol. Sci..

[45] Lee AG (2020). Spaceflight associated neuro-ocular syndrome (SANS) and the neuro-ophthalmologic effects of microgravity: a review and an update. npj Microgravity.

[46] Tanqueray EM, Eusuf D, England EL, Pinder AR, Shelton C (2020). Airway management in space: a novice skill?. Br. J. Anaesth..

[47] Wang HE (2012). Endotracheal intubation versus supraglottic airway insertion in out-of-hospital cardiac arrest. Resuscitation.

[48] Benoit JL, Gerecht RB, Steuerwald MT, McMullan JT (2015). Endotracheal intubation versus supraglottic airway placement in out-of-hospital cardiac arrest: a meta-analysis. Resuscitation.

[49] Wang CH (2020). Comparing effectiveness of initial airway interventions for out-of-hospital cardiac arrest: a systematic review and network meta-analysis of clinical controlled trials. Ann. Emerg. Med..

[50] Wetsch WA (2015). In a difficult access scenario, supraglottic airway devices improve success and time to ventilation. Eur. J. Emerg. Med..

[51] Benger JR (2018). Effect of a strategy of a supraglottic airway device vs tracheal intubation during out-of-hospital cardiac arrest on functional outcome: the AIRWAYS-2 randomized clinical trial. JAMA.

[52] Thornton WE, Linder BJ, Moore TP, Pool SL (1987). Gastrointestinal motility in space motion sickness. Aviat. Space Environ. Med..

[53] Mohr S (2013). Developing the skill of laryngeal mask insertion. Anaesthesist.

[54] Gellerfors M (2018). Pre-hospital advanced airway management by anaesthetist and nurse anaesthetist critical care teams: a prospective observational study of 2028 pre-hospital tracheal intubations. Br. J. Anaesth..

[55] Kuypers MI (2013). Emergency and wilderness medicine training for physician astronauts on exploration class missions. Wild. Env. Med..

[56] Wetsch WA (2012). Comparison of different video laryngoscopes for emergency intubation in a standardized airway manikin with immobilized cervical spine by experienced anaesthetists. A randomized, controlled crossover trial. Resuscitation.

[57] Harve-Rytsala H, Paal P, Kurola J (2021). To the Moon and beyond-Pushing boundaries in critical emergency medicine. Acta Anaesthesiol. Scand..

[58] Cavus E, Byhahn C, Dörges V (2017). Classification of videolaryngoscopes is crucial. Br. J. Anaesth..

[59] Wallace CD, Foulds LT, McLeod GA, Younger RA, McGuire BE (2015). A comparison of the ease of tracheal intubation using a McGrath MAC (®) laryngoscope and a standard Macintosh laryngoscope. Anaesthesia.

[60] Wetsch WA (2013). Tracheal intubation in the ice-pick position with video laryngoscopes: a randomised controlled trial in a manikin. Eur. J. Anaesthesiol..

[61] Zraier S, Bloc S, Chemit M, Amathieu R, Dhonneur G (2014). Intubation in the operating theatre using the Video-Airtraq laryngoscope in difficult circumstances by a face-to-face tracheal intubation technique. Br. J. Anaesth..

[62] Amathieu R (2012). Simulating face-to-face tracheal intubation of a trapped patient: a randomized comparison of the LMA Fastrach^TM^, the GlideScope^TM^, and the Airtraq^TM^ laryngoscope. Br. J. Anaesth..

[63] Dhonneur G, Zraier S, Sebbah JL, Haouache H (2014). Urgent face-to-face tracheal re-intubation using Video-Airtraq in ICU patients placed in the sitting position. Intensive Care Med..

[64] Aziz M (2016). Success of Intubation Rescue Techniques after Failed Direct Laryngoscopy in Adults: A Retrospective Comparative Analysis from the Multicenter Perioerative Outcomes Group. Anesthesiology.

[65] Teichman P, Donchin Y, Kot R (2007). International Aeromedical Evacuation. N. Engl. J. Med..

[66] Stepaniak, P. Hamilton, G. C. Stizza, D. Garrison, R. Gerstner, D. Considerations for medical transport from the Space Station via an Assured Crew Return Vehicle (ACRV). =https://ntrs.nasa.gov/search.jsp?R=20010073453. Published (2001).

[67] Hodkinson PD, Anderton RA, Posselt BN, Fong KJ (2017). An overview of space medicine. Br. J. Anaesth..

[68] Kirkpatrick AW (2017). Damage control surgery in weightlessness: a comparative study of simulated torso hemorrhage control comparing terrestrial and weightless conditions. J. Trauma Acute Care Surg..

[69] Drudi L, Ball CG, Kirkpatrick AW, Saary J, Grenon SM (2012). Surgery in Space: where are we at now?. Acta Astronaut.

[70] Kirkpatrick AW (2009). Severe traumatic injury during long duration spaceflight: Light years beyond ATLS. J. Trauma. Manag. Outcomes.

[71] Wang X (2018). An original design of remote robot-assisted intubation system. Sci. Rep..

[72] Boehler, Q. et al. “REALITI: A Robotic Endoscope Automated via Laryngeal Imaging for Tracheal Intubation,” in IEEE Transactions on Medical Robotics and Bionics, vol. 2, no. 2, pp. 157–164, May 10.1109/TMRB.2020.2969291 (2020).

[73] Komorowski M, Fleming S (2015). Intubation after rapid sequence induction performed by non-medical personnel during space exploration missions: a simulation pilot study in a Mars analogue environment. Extrem. Physiol. Med..

[74] Bernard CI, Thierry S, Morineau T (2021). Turing machine task analysis: specifying emergency assistance functions for a telemedicine system. Cogn. Tech. Work.

[75] Koetter KP, Maleck WH (1997). Reference for ice-pick position for intubation. Prehosp. Emerg. Care..

[76] Venezia D, Wackett A, Remedios A, Tarsia V (2012). Comparison of sitting face-to-face intubation (two-person technique) with standard oral tracheal intubation in novices: a mannequin study. J. Emerg. Med..

[77] Schober P, Krage R, van Groeningen D, Loer SA, Schwarte LA (2014). Inverse intubation in entrapped trauma casualties: a simulator based, randomised cross-over comparison of direct, indirect and video laryngoscopy. Emerg. Med. J..

[78] Anglin C, Wyss UP, Pichora DR (2000). Glenohumeral contact forces. Proc. Inst. Mech. Eng. H..

[79] Klemt C (2018). Analysis of shoulder compressive and shear forces during functional activities of daily life. Clin. Biomech..

[80] Hinkelbein J, Russomano T, Hinkelbein F, Komorowski M (2018). Cardiac arrest during space missions: specificities and challenges. Trends Anaesth. Crit. Care.

[81] Hinkelbein J, Komorowski M, Grau S (2018). Effects of Spaceflight on Astronaut Brain Structure: The potential impact of variable time-points in analysis of brain structure after spaceflight. N. Engl. J. Med..

