# Supplementary material for: Evaluation of free-floating tracheal intubation in weightlessness via ice-pick position with a direct laryngoscopy and classic approach with indirect videolaryngoscopy
Source: NPJ Microgravity. 2023 Sep 8;9:73. doi: 10.1038/s41526-023-00314-y (PMC10491756; doi:10.1038/s41526-023-00314-y)
Supplement: Supplementary file 1 — Reporting Summary [file 41526_2023_314_MOESM1_ESM.pdf]

nature portfolio

Corresponding author(s): NPJMGRAV-00783

Last updated by author(s): Apr 2, 2022

Reporting Summary

Nature Portfolio wishes to improve the reproducibility of the work that we publish. This form provides structure for consistency and transparency in reporting. For further information on Nature Portfolio policies, see our Editorial Policies and the Editorial Policy Checklist.

Statistics

For all statistical analyses, confirm that the following items are present in the figure legend, table legend, main text, or Methods section.

n/a	Confirmed	
☐	☒	The exact sample size (n) for each experimental group/condition, given as a discrete number and unit of measurement
☐	☒	A statement on whether measurements were taken from distinct samples or whether the same sample was measured repeatedly
☐	☒	The statistical test(s) used AND whether they are one- or two-sided<br>*Only common tests should be described solely by name; describe more complex techniques in the Methods section.*
☐	☒	A description of all covariates tested
☐	☒	A description of any assumptions or corrections, such as tests of normality and adjustment for multiple comparisons
☐	☒	A full description of the statistical parameters including central tendency (e.g. means) or other basic estimates (e.g. regression coefficient) AND variation (e.g. standard deviation) or associated estimates of uncertainty (e.g. confidence intervals)
☐	☒	For null hypothesis testing, the test statistic (e.g. F, t, r) with confidence intervals, effect sizes, degrees of freedom and P value noted<br>*Give P values as exact values whenever suitable.*
☒	☐	For Bayesian analysis, information on the choice of priors and Markov chain Monte Carlo settings
☒	☐	For hierarchical and complex designs, identification of the appropriate level for tests and full reporting of outcomes
☐	☒	Estimates of effect sizes (e.g. Cohen's d, Pearson's r), indicating how they were calculated

Our web collection on statistics for biologists contains articles on many of the points above.

Software and code

Policy information about availability of computer code

Data collection	Data was collected with Excel V16.43
Data analysis	Data was processed with Python (library Statsmodels).

For manuscripts utilizing custom algorithms or software that are central to the research but not yet described in published literature, software must be made available to editors and reviewers. We strongly encourage code deposition in a community repository (e.g. GitHub). See the Nature Portfolio guidelines for submitting code & software for further information.

Data

Policy information about availability of data

All manuscripts must include a data availability statement. This statement should provide the following information, where applicable:

- Accession codes, unique identifiers, or web links for publicly available datasets
- A description of any restrictions on data availability
- For clinical datasets or third party data, please ensure that the statement adheres to our policy

All relevant data are available from the first author (S.T) on request.

Field-specific reporting

Please select the one below that is the best fit for your research. If you are not sure, read the appropriate sections before making your selection.

☒ Life sciences ☐ Behavioural & social sciences ☐ Ecological, evolutionary & environmental sciences

For a reference copy of the document with all sections, see nature.com/documents/nr-reporting-summary-flat.pdf

Life sciences study design

All studies must disclose on these points even when the disclosure is negative.

Sample size	The sample size was dictated by the limited number of parabolas available, and the number of conditions to be tested (two).
Data exclusions	No data exclusion was reported in this studt
Replication	N/A
Randomization	No previous randomization was performed. Data was analyzed with generalized linear mixed-effects models (GLMM); it is an extension to the generalized linear model (GLM) which takes into account random effects. These models are traditionally used for longitudinal data, such as measurements within successive parabolas. The choice of this model is based on the possible learning effect that applied to each subject during the in-flight intubation sequences. Repeating procedures exposes subjects to learning that excludes any hypothesis of independence between successive measurements, regardless of the intubation technique used
Blinding	No binding was possible due to the nature of the study.

Reporting for specific materials, systems and methods

We require information from authors about some types of materials, experimental systems and methods used in many studies. Here, indicate whether each material, system or method listed is relevant to your study. If you are not sure if a list item applies to your research, read the appropriate section before selecting a response.

Materials & experimental systems

n/a	Involved in the study	
☒	☐	Antibodies
☒	☐	Eukaryotic cell lines
☒	☐	Palaeontology and archaeology
☒	☐	Animals and other organisms
☐	☒	Human research participants
☐	☒	Clinical data
☒	☐	Dual use research of concern

Methods

n/a	Involved in the study	
☒	☐	ChIP-seq
☒	☐	Flow cytometry
☒	☐	MRI-based neuroimaging

Human research participants

Policy information about studies involving human research participants

Population characteristics	Nine trained subjects (seven with daily practice of intubation and two non-doctors but with an intubation experience of over 20 procedures in a simulation center) participated in the experiment. All participants were volunteers and provided written informed consent for the research
Recruitment	Participants were recruited in the proncipal investigation's professional circle.
Ethics oversight	CNES Centre National d'Etudes Spatiales.

Note that full information on the approval of the study protocol must also be provided in the manuscript.

Clinical data

Policy information about clinical studies

All manuscripts should comply with the ICMJE guidelines for publication of clinical research and a completed CONSORT checklist must be included with all submissions.

Clinical trial registration	NCT05303948
Study protocol	Study protocol can be found on ClinicalTrials.gov

Data collection	Data was collected by an assistant operator during the flight after each attempt on a paper checklist, then reported on a computer after the flight.
Outcomes	Bi-pulmonary ventilation success was assessed by the intubating operator performing chest auscultation and by the measurement of a tidal volume on the mannequin’s electronic sensors. Selective side intubation was considered as failure. The duration of each attempt was recorded and ended with vocal confirmation of tube placement from the intubating operator or at the end of the parabola. Finally, a subjective score of confidence in the correct tube placement was recorded after each attempt and ranked from minus ten (complete certainty about failure) to plus ten (complete certainty about success). A score of zero was pronounced by the operator when he/she had maximum uncertainty about success or failure.
